# Supplementary material for: Are there lane advantages in track and field?
Source: PLoS One. 2022 Aug 3;17(8):e0271670. doi: 10.1371/journal.pone.0271670 (PMC9348673; doi:10.1371/journal.pone.0271670)
Supplement: S1 File — (ZIP) [file pone.0271670.s001.zip › READ ME.rtf]

This is a replication package for:“Are there lane advantages in track and field?” by David MunroThe following disclaimers hold:The data provided is for general informational purposes only. All information is provided in good faith, however I make no representation or warranty of any kind, express or implied, regarding the accuracy, adequacy, validity, reliability, availability or completeness of any information herein.The data for this analysis was collected from:https://worldathletics.org/competitions/If you use this data or replication package in an academic capacity please cite the following working paper:Munro, David. "Are there lane advantages in track and field?." Available at SSRN 3801883 (2021).Or, hopefully, the published version of the paper..Notes about the data:It includes data from the World Championships and U20 World Championships from 2000-2019 and the following important points are worth highlighting:1) In recent years, the 100m event can have a “Preliminary Round” prior to Round 1. It appears in the data that the winners of the preliminary round are sorted into the outside lanes of the Round 1 heats. As such, the Round 1 lane assignments are non-random. Because of this, when there exists a Preliminary Round, I use only this data and not the Round 1 data.2) Certain events are occasionally missing startlist data, which is required to know a runners’ lane assignment. These include: -2011 World Championship Womens 400m -2003 World Championship Mens 100m-2010 U20 Womens 800m-2008 U20 Womens 800mSince the analysis in the paper depends on knowing lane assignments, these events are excluded from the data.3) In rare cases, in the 800m, two runners are assigned to lane 8. These runners are excluded from the data.4) Data on personal bests and seasonal bests are central in the analysis, and runners are occasionally missing this data. Runners with this missing data are excluded from the data.
