# Supplementary material for: Are there lane advantages in track and field?
Source: PLoS One. 2022 Aug 3;17(8):e0271670. doi: 10.1371/journal.pone.0271670 (PMC9348673; doi:10.1371/journal.pone.0271670)
Supplement: S1 Appendix — (PDF) [file pone.0271670.s002.pdf]

Supporting information

S1 Appendix. Additional Empirical Results

This section includes the baseline randomization checks for the 200m, 400m, and 800m as well as some additional randomization checks conducted via probit regressions.

Probit Regressions

As an additional check on the randomization across lanes, I examine whether the probability a runner is assigned to a lane is correlated with their prior performance (season's best). To do this, I estimate the following statistical model:

$$Pr(T_k = 1|X) = \Phi(\alpha_0 + \beta_1 X) \tag{8}$$

Which examines how the probability of being assigned to a given lane k depends on a runner's ability (season's best), which is denoted by X. If random assignment is successful, there should be no relationship between the probability of being assigned to a lane and a runner's season's best. This analysis is conducted separately for the 100, 200, 400, and 800m and the results are reported in Table 12, Table 13, Table 14, and Table 15, respectively.

Table 9. Randomization check for 200m

Coeff. (Ind. Var.)	Mens		Womens		Pooled	
β_1 (Lane 1)	0.2595**	0.1648*	0.0221	0.0473	0.1762*	0.1264*
	(0.1052)	(0.0846)	(0.1936)	(0.1324)	(0.0958)	(0.0719)
	[54]	[49]	[23]	[22]	[77]	[71]
β_3 (Lane 3)	0.0409	-0.0012	-0.1058	-0.0804	-0.0234	-0.0361
	(0.0743)	(0.0687)	(0.1263)	(0.0967)	(0.0692)	(0.0571)
	[119]	[110]	[94]	[88]	[213]	[198]
β_4 (Lane 4)	0.0085	-0.0323	-0.0758	-0.0542	-0.0283	-0.0418
	(0.0718)	(0.0638)	(0.1408)	(0.0968)	(0.0738)	(0.0554)
	[120]	[112]	[96]	[91]	[216]	[203]
β_5 (Lane 5)	-0.0446	-0.0420	-0.1151	-0.0096	-0.0753	-0.0277
	(0.0683)	(0.0644)	(0.1173)	(0.1003)	(0.0640)	(0.0566)
	[123]	[119]	[96]	[92]	[219]	[211]
β_6 (Lane 6)	-0.0154	-0.0268	-0.1032	-0.0016	-0.0537	-0.0162
	(0.0769)	(0.0682)	(0.1201)	(0.1032)	(0.0678)	(0.0587)
	[123]	[119]	[91]	[87]	[214]	[206]
β_7 (Lane 7)	-0.0311	-0.0309	-0.1865*	-0.0355	-0.1000†	-0.0325
	(0.0720)	(0.0677)	(0.1121)	(0.0960)	(0.0637)	(0.0570)
	[118]	[114]	[98]	[97]	[216]	[211]
β_8 (Lane 8)	0.0679	0.0784	0.0349	0.0340	0.0538	0.0593
	(0.0682)	(0.0636)	(0.1452)	(0.1000)	(0.0743)	(0.0561)
	[119]	[115]	[94]	[88]	[213]	[203]
β_9 (Lane 9)	0.1248	-0.0027	-0.0364	-0.0082	0.0862	-0.0050
	(0.1463)	(0.1044)	(0.1782)	(0.1330)	(0.1131)	(0.0823)
	[34]	[32]	[27]	[24]	[62]	[56]
α_1 (Male)					-2.659***	-2.611***
					(0.0379)	(0.0305)
	[34]	[32]	[27]	[24]	[62]	[56]
α_0 (constant)	20.85***	20.81***	23.59***	23.43***	23.54***	23.42***
	(0.0499)	(0.0475)	(0.0896)	(0.0684)	(0.0553)	(0.0446)
N	926	880	708	671	1634	1551
R^2	0.0152	0.0120	0.0069	0.0030	0.7698	0.8370
Outliers Removed	No	Yes	No	Yes	No	Yes
F-stat.	1.49	1.40	0.69	0.27	1.77	1.23
p-value	0.1577	0.1945	0.6987	0.9757	0.0792	0.2790

Table notes: This table reports the coefficients estimated from model Eq (5). To ease interpretation of the results, the independent variable associated with each coefficient estimate is highlighted in parentheses. The number of observations per lane are reported in square brackets. Robust standard errors are reported in parentheses. †, *, **, and *** denote significance at the one-sided 10%, two-sided 10%, 5%, and 1% levels, respectively.

Table 10. Randomization check for 400m

Coeff. (Ind. Var.)	Mens		Womens		Pooled	
β_1 (Lane 1)	0.2277	0.0816	-0.0052	-0.0355	0.1386	0.0354
	(0.2774)	(0.2130)	(0.3337)	(0.3344)	(0.2139)	(0.1843)
	[49]	[46]	[31]	[30]	[80]	[76]
β_3 (Lane 3)	-0.0416	-0.0550	0.5299*	0.1842	0.1954	0.0417
	(0.1759)	(0.1508)	(0.2918)	(0.2621)	(0.1592)	(0.1387)
	[118]	[112]	[85]	[76]	[203]	[188]
β_4 (Lane 4)	-0.0935	-0.1264	-0.0402	-0.2114	-0.0746	-0.1633
	(0.1790)	(0.1466)	(0.2474)	(0.2379)	(0.1466)	(0.1306)
	[119]	[114]	[93]	[87]	[212]	[201]
β_5 (Lane 5)	0.0524	0.0437	0.0195	-0.1687	0.0306	-0.0539
	(0.1733)	(0.1469)	(0.2678)	(0.2519)	(0.1524)	(0.1372)
	[112]	[106]	[95]	[90]	[207]	[196]
β_6 (Lane 6)	0.0170	-0.0663	0.1065	-0.1163	0.0514	-0.0892
	(0.1877)	(0.1553)	(0.2588)	(0.2325)	(0.1568)	(0.1326)
	[111]	[105]	[93]	[88]	[204]	[193]
β_7 (Lane 7)	0.0107	-0.1092	0.0529	0.0204	0.0244	-0.0508
	(0.1877)	(0.1464)	(0.2387)	(0.2381)	(0.1474)	(0.1314)
	[114]	[107]	[91]	[88]	[205]	[195]
β_8 (Lane 8)	-0.0346	-0.0553	0.2018	0.0224	0.0655	-0.0204
	(0.1845)	(0.1541)	(0.2865)	(0.2536)	(0.1619)	(0.1395)
	[107]	[101]	[86]	[83]	[193]	[184]
β_9 (Lane 9)	0.0365	-0.1543	-0.0300	-0.2148	-0.0052	-0.1847
	(0.2988)	(0.2284)	(0.3650)	(0.2977)	(0.2326)	(0.1834)
	[33]	[30]	[31]	[30]	[64]	[60]
α_1 (Male)					-6.346***	-6.329***
					(0.0854)	(0.0739)
α_0 (constant)	46.34***	46.22***	52.58***	52.54***	52.64***	52.54***
	(0.1216)	(0.1017)	(0.1738)	(0.1724)	(0.1161)	(0.1059)
N	872	826	677	643	1549	1469
R^2	0.0025	0.0032	0.0091	0.0068	0.7922	0.8450
Outliers Removed	No	Yes	No	Yes	No	Yes
F-stat.	0.62	0.48	0.23	0.33	0.42	0.48
p-value	0.7585	0.8712	0.9864	0.9546	0.9097	0.8741

Table notes: This table reports the coefficients estimated from model Eq (5). To ease interpretation of the results, the independent variable associated with each coefficient estimate is highlighted in parentheses. The number of observations per lane are reported in square brackets. Robust standard errors are reported in parentheses. †, *, **, and *** denote significance at the one-sided 10%, two-sided 10%, 5%, and 1% levels, respectively.

Table 11. Randomization check for 800m

Coeff. (Ind. Var.)	Mens		Womens		Pooled	
β_1 (Lane 1)	-0.1889	0.4004	-0.2233	-0.3843	-0.1657	0.1235
	(0.5907)	(0.4276)	(0.6859)	(0.6804)	(0.4519)	(0.3760)
	[51]	[49]	[31]	[29]	[82]	[78]
β_3 (Lane 3)	-0.7940*	-0.0088	0.8690	0.1662	-0.0855	0.0645
	(0.4778)	(0.3324)	(0.7144)	(0.5012)	(0.4190)	(0.2872)
	[103]	[101]	[86]	[82]	[189]	[183]
β_4 (Lane 4)	-0.6156	-0.3734	0.9703	0.8392	0.0564	0.1720
	(0.5549)	(0.3296)	(0.8560)	(0.8608)	(0.4931)	(0.4274)
	[103]	[98]	[85]	[82]	[188]	[180]
β_5 (Lane 5)	-0.3730	0.2005	0.6786	0.1593	0.0580	0.1797
	(0.4784)	(0.3205)	(0.6236)	(0.5521)	(0.3862)	(0.3000)
	[107]	[101]	[84]	[79]	[191]	[180]
β_6 (Lane 6)	-0.4019	0.3070	0.1574	-0.0529	-0.1782	0.1622
	(0.4877)	(0.3370)	(0.6264)	(0.5652)	(0.3889)	(0.3076)
	[108]	[105]	[79]	[74]	[187]	[179]
β_7 (Lane 7)	-0.2732	0.0149	0.6772	0.3588	0.1142	0.1633
	(0.5382)	(0.3244)	(0.6058)	(0.5330)	(0.4051)	(0.2948)
	[106]	[99]	[81]	[78]	[187]	[177]
β_8 (Lane 8)	-0.3640	-0.0287	1.477*	0.3521	0.4037	0.1334
	(0.5112)	(0.3121)	(0.8342)	(0.5573)	(0.4626)	(0.2959)
	[97]	[90]	[72]	[65]	[169]	[155]
β_9 (Lane 9)	-0.8978†	-0.3632	1.431	1.474	0.1280	0.4825
	(0.6474)	(0.4462)	(2.158)	(2.233)	(1.062)	(1.063)
	[32]	[31]	[28]	[27]	[60]	[58]
α_1 (Male)					-15.54***	-15.46***
					(0.2479)	(0.209)
α_0 (constant)	107.92***	107.05***	122.36***	122.25***	123.01***	122.39***
	(0.4084)	(0.2279)	(0.3826)	(0.370)	(0.317)	(0.245)
N	803	762	613	582	1416	1344
R^2	0.0055	0.0096	0.0079	0.0073	0.7568	0.8238
Outliers Removed	No	Yes	No	Yes	No	Yes
F-stat.	0.83	0.37	0.62	0.86	0.28	0.09
p-value	0.5749	0.9374	0.7611	0.5499	0.9715	0.9995

Table notes: This table reports the coefficients estimated from model Eq (5). To ease interpretation of the results, the independent variable associated with each coefficient estimate is highlighted in parentheses. The number of observations per lane are reported in square brackets. Robust standard errors are reported in parentheses. †, *, **, and *** denote significance at the one-sided 10%, two-sided 10%, 5%, and 1% levels, respectively.

Table 12. Probit randomization check for 100m

Coeff. (Ind. Var.)	Lane 1	Lane 2	Lane 3	Lane 4	Lane 5	Lane 6	Lane 7	Lane 8	Lane 9
β_1 (SB)	-0.079	0.0348	0.0459	-0.0642	0.0016	-0.0018	0.0407	0.0019	-0.0297
	(0.0633)	(0.0518)	(0.0517)	(0.0536)	(0.0518)	(0.0513)	(0.0525)	(0.0538)	(0.0808)
α_0 (constant)	-0.600	-1.530***	-1.665***	-0.427	-1.136**	-1.093*	-1.586***	-1.186**	-1.472*
	(0.699)	(0.576)	(0.575)	(0.593)	(0.575)	(0.569)	(0.584)	(0.597)	(0.895)
N	1671	1671	1671	1671	1671	1671	1671	1671	1671
R^2	0.0019	0.0004	0.0006	0.0011	0.0000	0.0000	0.0006	0.0000	0.0003

Table notes: Additional randomization check for the 100m. This table reports the coefficients estimated from model Eq (8). Robust standard errors are reported in parentheses. †, *, **, and *** denote significance at the one-sided 10%, two-sided 10%, 5%, and 1% levels, respectively.

Table 13. Probit randomization check for 200m

Coeff. (Ind. Var.)	Lane 1	Lane 2	Lane 3	Lane 4	Lane 5	Lane 6	Lane 7	Lane 8	Lane 9
β_1 (SB)	-0.0405	0.0096	-0.0071	0.0049	-0.0043	-0.0059	-0.0173	0.021	0.0381
	(0.0358)	(0.0255)	(0.0252)	(0.0248)	(0.0251)	(0.0251)	(0.0254)	(0.0248)	(0.0352)
α_0 (constant)	-0.801	-1.375**	-0.967*	-1.218**	-1.02*	-0.992*	-0.736†	-1.577***	-2.594***
	(0.785)	(0.563)	(0.556)	(0.549)	(0.553)	(0.555)	(0.560)	(0.547)	(0.783)
N	1777	1777	1777	1777	1777	1777	1777	1777	1777
R^2	0.002	0.0001	0.0001	0.0011	0.0000	0.0000	0.0003	0.0005	0.0019

Table notes: Additional randomization check for the 200m. This table reports the coefficients estimated from model Eq (8). Robust standard errors are reported in parentheses. †, *, **, and *** denote significance at the one-sided 10%, two-sided 10%, 5%, and 1% levels, respectively.

Table 14. Probit randomization check for 400m

Coeff. (Ind. Var.)	Lane 1	Lane 2	Lane 3	Lane 4	Lane 5	Lane 6	Lane 7	Lane 8	Lane 9
β_1 (SB)	-0.0109	-0.0124	0.0060	-0.0026	0.0021	0.0016	-0.0031	0.0051	0.0158
	(0.0150)	(0.0115)	(0.0109)	(0.0110)	(0.0111)	(0.0110)	(0.0110)	(0.0111)	(0.0149)
α_0 (constant)	-1.12†	-0.577	-1.41***	-0.988*	-1.22**	-1.20**	-0.955†	-1.39**	-2.47***
	(0.738)	(0.566)	(0.537)	(0.543)	(0.545)	(0.543)	(0.542)	(0.545)	(0.740)
N	1645	1645	1645	1645	1645	1645	1645	1645	1645
R^2	0.0008	0.0001	0.0002	0.0000	0.0000	0.0000	0.0001	0.0002	0.0018

Table notes: Additional randomization check for the 400m. This table reports the coefficients estimated from model Eq (8). Robust standard errors are reported in parentheses. †, *, **, and *** denote significance at the one-sided 10%, two-sided 10%, 5%, and 1% levels, respectively.

Table 15. Probit randomization check for 800m

Coeff. (Ind. Var.)	Lane 1	Lane 2	Lane 3	Lane 4	Lane 5	Lane 6	Lane 7	Lane 8	Lane 9
β_1 (SB)	-0.0080	-0.0017	-0.0002	0.0017	-0.0001	-0.0029	0.0011	0.0038	0.0037
	(0.0063)	(0.0049)	(0.0046)	(0.0045)	(0.0047)	(0.0047)	(0.0046)	(0.0046)	(0.006)
α_0 (constant)	-0.69	-1.01*	-1.10**	-1.31**	-1.10**	-0.794†	-1.227**	-1.60***	-2.12***
	(0.722)	(0.563)	(0.528)	(0.520)	(0.533)	(0.541)	(0.527)	(0.533)	(0.695)
N	1490	1490	1490	1490	1490	1490	1490	1490	1490
R^2	0.0025	0.0001	0.0000	0.0001	0.0000	0.0003	0.0000	0.0006	0.0007

Table notes: Additional randomization check for the 800m. This table reports the coefficients estimated from model Eq (8). Robust standard errors are reported in parentheses. †, *, **, and *** denote significance at the one-sided 10%, two-sided 10%, 5%, and 1% levels, respectively.
