# Supplementary material for: Are there lane advantages in track and field?
Source: PLoS One. 2022 Aug 3;17(8):e0271670. doi: 10.1371/journal.pone.0271670 (PMC9348673; doi:10.1371/journal.pone.0271670)
Supplement: Attachment — Submitted filename: Referee Report_PONE-D-21-35820.pdf [file pone.0271670.s004.pdf]

Referee Report for the manuscript PONE-D-21-35820R1
"Are there lane advantages in track and field?"

The paper focuses on the common belief about the fact that some lanes on the track, in particular the middle ones such as 3-6, are advantageous with respect to the others. Using a sample of random assigned lanes in the first round of events the author finds no evidence supporting the common belief and, in some cases, even contrary. The work concludes that the common belief is a folk tale.

General comments

The paper answers an interesting and "fanciful" question about an alleged benefit in racing in the middle lanes. The paper is well motivated and the results are likely to have a great echo in non-academic fields. The econometric analysis is correctly conducted, and the results obtained seem sound. Notwithstanding, there are some points that deserve to be refined. In particular:

1) notation of eq (1) and (2). As far as I've understood, the dummies L_k represent the $Lane_k$ for runner *i* in heat *j* and their value is supposed to change according to the runner and to the heat. Therefore, they should also report the subscript ij and the summation is up to k-1 given the collinearity problem.
2) When the pulled sample is used a gender dummy should be included
3) Quoting from page 7 "*only lane 1 has a statistically significant relationship with SB, which, again, is likely an effect of the low number of observations*". It is difficult to understand this claim, it seems the other way round. When the number of observations increases, the standard error (SE) decreases, and consequently the *t*-stat increases as t=beta/SE(beta), therefore when the number of observations is low one over accepts the null of non significant statistical effects, i.e. weak power. I think the claim should be revised and another piece of explanation for that significance should be put forth.
4) Power problems. I wonder whether there are other tests that can be implemented to analyse the issue whether the results are driven by low power or whether they can be read as pure lack of statistical significance. I am not an expert of this specific filed, but one idea could be to adapt the tests proposed by Cattaneo Titiunik and Vazquez-Bera (2019) to the case under scrutiny.

Minor issues

The references must be reported according to the common practice followed in the literature. In the current version, no contribution reports the year.

References

Cattaneo M-D., Titiunik R., and Vazquez-Bera G., 2019. Power calculations for regression-discontinuity designs. *The Stata Journal,* 19(1): 210-245.
