# Supplementary material for: Are there lane advantages in track and field?
Source: PLoS One. 2022 Aug 3;17(8):e0271670. doi: 10.1371/journal.pone.0271670 (PMC9348673; doi:10.1371/journal.pone.0271670)
Supplement: Attachment — Submitted filename: Reviewer 2 Comments- RR2.pdf [file pone.0271670.s006.pdf]

Response to Reviewers:

Reviewer #2: The topic of the paper is undoubtedly appealing and interesting. However, in this third round of revision, my concerns remain the same, since most of my major comments/suggestions to improve the paper are again not taken fully and/or appropriately into account. To this end, in what follows, once again I report almost the same major comments present in my two previous reports.

Major comments:
1. The general description of the statistical methodology applied in the paper, as well as the relevant literature in this field are still completely missing. I still kindly suggest to the author to carefully describe in details in a separate section and/or subsection the main statistical theory (i.e., the statistical methodology used in this paper) in a general and rigorous way, i.e., main formulas, assumptions and so on. Moreover, the most relevant literature in the causal inference framework, which is still completely missing, should be also reported by the author and cited where necessary. Regarding the statistical models in formula (1) and (2), they are still reported inaccurately: again, why 〖Time〗_(i,j) is used to indicate the response variable? Statistical models should be reported accurately in general, for instance using y_ij for the response variable. Similar issues also apply to the independent variables included in the model. Again, what about the subscripts: i=1,…?,j=1,…? That is, the subscripts "i" and "j" goes from 1 to what? About the subscript "k": why it goes from 1 to n? Why not to "K" for example, since the letter "n" in statistics is usually used to indicate the sample size. Also, I think it is confused and not in line with the basic statistical terminology to use always and everywhere "regression specification" only to refer to a statistical model.

Response: see response to 2.

2. In fact, it is well-known that in a causal inference framework with random assignment to treatment, one can use a regression model to estimate causal effects. But, please note that when one deals with classical linear regression modelling (NOT in a causal inference framework with the well-known assumptions on the treatment assignments mechanism), one cannot state that this is a causal effect, because correlation does not imply causality. I understood that the author deals with causal inference framework, but in my opinion, it should be accurately described in a separate section and/or subsection through a general description of the main statistical theory, as I already suggested at my previous major comment #1.

Response: I agree that without random assignment linear regression can't speak about causal effects. In relation to your comments 1 and 2 I have added a separate section detailing the causal inference framework, which highlights the importance of the independence assumption (treatment status independent of outcomes). This emphasizes the importance of utilizing the heats which implement random assignment to lanes.

As per your request, I have changed the notation to Y_{i,j} instead of "Time." The i and j notation is not over sums, so they are just denoting unique observations for different "i" and "j", so it is not necessary to stipulate the limits of these indexes. In relation to the 1 to n notation, I changed the notation in rewriting the causal inference section and no longer use "n."

As per your request, I have also changed "specification" to "model."

3. Please, justify and explain better your statement on the use of propensity score matching in your specific case-study. Furthermore, my previous question was not just limited to propensity score matching: what about other existing approaches in the literature to deal with this issue? The statements along the paper which rely on "low number of observations" are still very confused, and, at least in this current form, they do

not seem appropriate. This is because from what I see in all the tables, the values reported for "N" are high rather than low. Please, justify.

Response: I believe your comment is referring to propensity score matching in relation to the randomization checks. Normally, propensity score matching is a way to match individuals in the treatment and control groups based on similar covariates (we can reduce the dimensionality of this by summarizing similar individuals by their propensity scores.) This matching is typically done when there are concerns that individuals in the treatment/control groups are systematically different. In this sense, the matching part only really makes sense when propensities vary systematically conditional on treatment status. However, one can use the first step (estimating propensities) to see if the randomization successfully balanced treatment and control groups. I think this is what you may be asking for. In the appendix I have added probit regressions where I estimate how the probability of being assigned to a lane is a function of runner ability (season's best). If these treatment probabilities vary systematically by runner ability that would be concerning about the randomization. Thankfully, none of the regressions show that season's best is significantly related to treatment status. I hope I have interpreted your comment appropriately.

In relation to the sample sizes, what I'm trying to highlight is that readers should be cautious about any statistical significance being derived from small sample sizes. For example, in the Women's 100m randomization check, there are statistically significant results for lanes 1 and 9. However, the sample sizes in these lanes are less than 40, relative to around 100 in the other lanes. These are relatively small sample sizes. The concern is that with smaller sample sizes it's more likely to have Type-1 error (incorrectly rejecting the null of no effect) (Leppink et al. 2016.). This also relates to Type-M error (Gelman and Carlin, 2014). They emphasize that significant results from small sample sizes often overstate the magnitude of the true effect: "*The problem, though, is that if sample size is too small, in relation to the true effect size, then what appears to be a win (statistical significance) may really be a loss (in the form of a claim that does not replicate).*" **My point is that in lanes with small numbers of observations, while there are occasionally significant results, readers should be skeptical of the replicability of those results. I have attempted to clarify this in the text.**

4. Again, in my opinion, suitable models diagnostics should be performed in order to appropriately evaluate the estimated statistical models. Please, note that they are definitely not just limited to standard errors.

Response: There are several diagnostics/robustness checks related to the model already in the paper: R^2, F-statistics, removing outliers, robust standard errors, and three different statistical models. None of the results are sensitive to these different checks. It's also important to emphasize that the coefficients of interest are on indicator variables (treatment indicators), and there is no functional form assumed here. It may also be relevant to note that with random assignment, choosing the appropriate model is not really necessary for a causal interpretation of regression. For more discussion of this, see Chp. 3 in Angrist and Pischke (2009).

Without specific guidance on what diagnostic(s) you think would be valuable to add and why, I don't know how else to respond to this comment. I apologize.

5. Again, Tables no.1-no.8: in all the tables, the estimated coefficients are reported incorrectly as Lane 1, Lane 3, Wind, etc. For example, the author should to write $\hat{\beta}_1$ rather than Lane 1, $\hat{\beta}_3$ rather than Lane 3, $\hat{\alpha}_1$ rather than Wind, and so on. It could seem very straightforward to understand, it is how R, Stata reports them, but the problem is that this is not correct, it's not the norm and it's an error.

Response: I've changed the notation in the tables to be \beta_1 etc., as you have requested. However, as a search of papers in PLOS ONE will highlight, I do not think this is a standard practice when reporting regression results, at least in the fields I am familiar with. To ease interpretation in light of this change, I have also included the independent variable in parenthesis beside each coefficient, so readers know what each coefficient represents. I hope you find this to be a reasonable compromise.
