# Supplementary material for: Are there lane advantages in track and field?
Source: PLoS One. 2022 Aug 3;17(8):e0271670. doi: 10.1371/journal.pone.0271670 (PMC9348673; doi:10.1371/journal.pone.0271670)
Supplement: Attachment — Submitted filename: PLOS ONE Response to reviewer comments.pdf [file pone.0271670.s003.pdf]

Response to reviewer comments:

Reviewer 1:

Overall, this paper is interesting and mostly well executed. However, it sometimes lacks in motivation and a deeper analysis that tries to establish the root causes for the (non-)findings, which could be done with the present data. There are also a few issues in the presentation, with the potential for improvement.

Motivation
- The main scientific relevance of the research question – as it is presented in the intro - currently rest with how much one trusts the claims in the cited literature on biomechanics and physical factors. The author should make the significance of this literature more clear if this is intended.
- If not, the author should make the other motivation for this paper – and the results' potential wider implications - more clear.
- This applies in particular to the motivational/psychological factors at play.

Response: Thanks for these suggestions. I have added more discussion in the introduction of the relevance of the paper for the literature examining the performance effects of motivational/psychological factors.

Physics, psychics and measurement
- Given that not all 9 nine lanes are always filled and that each lane has its own average curvature (e.g., measured by radian covered per meter run and its concentration), could one not construct a related variable which measures and tests biomechanics argument directly (and continuously)?

Response: This is interesting. From the biomechanics literature there is no single "mechanism" that would slow runners down in tighter corners. Some ideas are that tight corners increase step frequency, lower foot force production, create asymmetries between legs, etc. Furthermore, while one can model the geometry of the track mathematically, to my knowledge there are not mathematical models relating this geometry to biomechanical factors. Without such a theory it's hard to know how to treat the effect of curvature. For example, is the curvature effect linear, non-linear, etc. What I like about the baseline regression specification is that it's agnostic about the lane-specific treatment effects. In relation to one of your points below, I have added two more specifications to the paper to explore the robustness of the results to other specifications.

- It should be made clear whether there are potentially opposing effect from other factors. For example Berger and Pope (2011) show that "being behind" spurs effort. People on inner tracks, if randomly assigned, are on average more often (literally) behind their opponents. Could this

be an explanation for null effects? (see also Teeselink, van den Assem and van Dolder, 2021 for an opposing account)?

Response: See next response.

- By constructing appropriate measures (e.g., whether someone was on the outmost lane and could not see competitors vs accounting for lane curvature which might not co-vary perfectly if not all lanes are filled) one could account for – and test - different theories.

Response: Thanks for these great comments. The evidence from the baseline specification is simply showing the net impact of being in a specific lane. I think this is ultimately what we care about in the track and field context, i.e. "are the middle lanes best?" But there is a subtle point you raise that the "being behind spurs effort" channel could still be active, but it's not strong enough to make the outside lanes slower. This question is more relevant for the motivational/psychological factors you highlight. Following your suggestion, I've added a separate analysis where I include an indicator variable for the outmost lane (which, as you note, is not perfectly correlated with lane number). I pursue this additional analysis in the 200 and 400m, where the staggered starts/effort effects would be most noticeable and find some evidence that the outermost lanes in the 200m do slow runners down. I find no statistical effect of the outermost lane in the 400m, which could be a product of noisier data. Anyway, thanks for these suggestions, I think they have really strengthened the paper.

- I was kind of surprised that wind but not wind shadow created by opponents – where lane assignment is probably relevant – was discussed.

Response: Thanks for this comment. I've never encountered it anecdotally from my participation in the sport and I searched for any discussion in track and field forums about wind shadows in sprint events and was unable to find anything (it's certainly a factor in long-distance events where runners draft, but I couldn't find anything in relation to sprinting). So, it seems like it's not a common thing that is highlighted in relation to lane assignments. I worry about adding discussion about this since it doesn't seem to be widely discussed, so I have left it out.

- What if the average skill athletes' level in the first round of these tournaments is not high enough that the finer features of the biomechanics and other subtle factors affecting performance just don't play out to be decisive? Maybe even the first round is highly challenging to get into and only achievable for pros, but without further contextual info that's hard to judge.

Response: I think this is an interesting question. My off-the-cuff response is that these are the world championships, so clearly these are the best runners in the world. I've tried to think of different ways to provide more contextual info, but nothing obvious came to mind… (e.g. look at average season's bests of this group of runners, but compare it to who?) Being the

world championships doesn't necessarily rule out that these lane effects are only/more salient for the cream of the crop, but I don't know if there is a way to assess that question reliably. Ultimately, exploiting the random assignment feature is important for obvious reasons, and this feature doesn't exist in the more elite rounds (e.g. semis/finals). I don't disagree with the possibility that lane effects would be more relevant for more elite athletes, but without a reliable way to empirically assess the question it feels very conjecture-y, so I have left this discussion out.

Econometric specification and presentation
- Are 8 lane dummies really the best specification, especially when there are concerns about statistical power? If effects are expected to be monotone across lanes, why not a continuous (linear or quadratic) function?

Response: See below.

- If, there is a good reason to not use a continuous specification, why can lanes not be grouped (e.g., into 1&2, 3&4, 5&6, 7&8) to achieve more power per coefficient?

Response: Thanks for these comments. In the baseline analysis I chose dummies for each lane to be as agnostic as possible regarding the functional form of any lane effects. As additional robustness checks, and to improve statistical power, I have added additional results where I group 1&2, 3&4, 5&6, 7&8&9 and where I treat lanes as a continuous variable. The general conclusions from the original baseline specification are also reflected in these additional results.

- Reading essentially 5 times the same specification and table but with different data sets is kind of hard and makes it hard to compare findings. Why not only present the 100m table as an example in the main text and then depict the (relevant) line coefficient and their SE graphically (e.g., lanes on the x-axis, normalised coefficients on the y-axis with SE and a line connecting them). This graphical presentation could then be added for all other distance to the same graph (e.g. by stacking the connecting lines with the coefficients). All other tables and the randomness check can then go to an appendix and the paper would be much more comprehensive. (Ideally the graph would also depict a normalised interval for MDE for each line.)

Response: I agree that there are a lot of tables to look at. I've moved the randomness checks for the 200, 400, and 800m to the appendix. I've also added a graphical depiction of the main results. Stacking everything in one graph became visually very cluttered. So, what I've done is to graphically display the estimated lane effects (from pooling men and women and excluding outliers) together with 95% confidence intervals in side-by-side figures for the 100, 200, 400 and 800m. I think this graphical display helps to interpret the results. I've left the regression result tables in the main text as I refer to them frequently in the text and it would be burdensome for readers to flip back and forth between the appendix.

Other (some minor, some not) issues

- “was put in place during in the 1985-86 rules under rule 141.11” reads like the rule were only in place in the years/season 1985/1986 while elsewhere we find “AAF World Championships and U20 World Championships from 2000 to 2019”. I suggest to clarify this (also footnote 3 which is hard to read).
- In general, I would advise to describe a bit more clearly how/when/how long the random assignment was introduced. Right now it reads just like it was and then a bunch of technical details. Maybe that can be presented more story-like.

Response: I have re-worded the section to emphasize that the random assignment rule was initiated in the 1985-86 season and remains in place today. I wish I had more of a “rule history” (i.e. why were rules changed, etc.) to rely on to tell a story here, but unfortunately I’ve been unable to find that information anywhere.

- I would also advise to refer to measures in the flow of the text not by their variable names but by what they describe in order to improve readability.

Response: I’ve done this where appropriate.

- the variable SB is first described as “SB is the runner’s season best race time”, then as “ assigned lanes based on prior race results (proxied here by SB)”. Am I correct that the author tries to claim that results prior to race can be proxied by SB, e.g., the best result across the whole season?

Response: I have re-written this section to help clarify.

- The sum in the regression equation should index over only 8 dummies, not 9, as one lane is the baseline.

Response: thanks for point this out, I have made this change.

- I did not find the data in the paper or an appendix, except for a link to a sports website. I would expect the authors to share, with the manuscript, i) the original dataset used, including any outliers or incomplete data dropped for the actual analysis, ii) a short description on how it was generated, iii) the script(s) used to analyse and pre-process the data and to generate all tables/figures.

Response: My apologies for this. I’m used to providing replication packages during the publication process, not prior. While the data is publicly available in uncompiled form from the IAAF, I have now included a link to a replication package with the compiled data.

<u>Reviewer 2:</u>

The topic of the paper is undoubtedly interesting and appealing. However, I have very serious concerns on the validity of the results presented in the paper due to the very poor and

inaccurate description of the applied statistical methodology. Following, the details of the review are divided into major and minor comments.
Major comments:
1) The general description of the statistical methodology applied in the paper is completely missing. This issue does not allow to appropriately evaluate the validity of the results reported in the paper. The author should to carefully describe the applied statistical methodology in details in a separate section, by reporting in a rigorous way the main theory (formulas, assumptions, and the corresponding references). Furthermore, the specifications of the statistical models in formulas (1) and (2) are completely inaccurate; for instance, subscripts are missing in these formulas, the random components are just reported as "error" rather than through the well-known statistical notation, models' assumptions are completely missing.

Response: My apologies for this omission. In the field I'm in, it would be seen as unnecessarily/obvious to discuss the theory/assumptions underlying the regression analysis. With random assignment to treatment, the analysis is quite straight and amounts to reporting average treatment effects. To be as agnostic as possible about the structure of these effects I estimate them using dummy (0,1) variables for lanes in a regression that controls for the other covariates. I have added some discussion of this to clarify.

2) My main concern relates to the validity of the results reported in the paper. Since the description of the statistical methodology is completely missing, from what I see, it seems that the author apply a linear regression model? At the same time, the author claims along the entire manuscript to estimate "the causal effect of line assignments on race times". However, it is well-known that we cannot speak about a causal effect when considering the "classical" linear regression model. There are several specific approaches for causal inference, as for instance the potential outcomes framework, causal graphs and similar; however, nothing is mentioned in the paper on this point. The issue mentioned above is of crucial importance on the entire validity and interpretation of the results reported in this paper, and it should be carefully justified and explained in details.

Response: Respectfully, "However, it is well-known that we cannot speak about a causal effect when considering the "classical" linear regression model" is simply incorrect. There is nothing inherently problematic about using linear regression to estimate causal effects. The issue is whether the assumptions of OLS are met or not. In non-experimental data, it is unlikely that the assumptions are met. But the whole point of the paper is to leverage random assignment to ensure the exogeneity assumption *is* met. There is nothing wrong with using OLS to estimate average treatment effects with random assignment.

3) Statistical model diagnostics are completely missing. They should be performed and reported in order to appropriately evaluate the estimated statistical models.

Response: As is now clarified in the paper, there is no functional form assumed in estimating the average treatment effects of lanes. As a result, in terms of model diagnostics, there is

nothing to test regarding the functional form or homoscedasticity for the main variables of interest (lane effects). In response to the other reviewer's suggestions, I have also added results estimated from two different regression specifications.

4) Tables no.1-no.8: in all the tables, the estimated coefficients are reported incorrectly as Lane 1, Lane 3, Wind, etc. For example, the author should to write $\hat{\beta}_1$ rather than Lane 1, $\hat{\beta}_3$ rather than Lane 3, $\hat{\alpha}_1$ rather than Wind, and so on. Moreover, R^2 are erroneously reported as "R ^ 2", and furthermore they should be discussed.

Response: Thanks for this comment. I have raised "2" to a superscript in "R^2". For readability sake, I feel "Lane 1" is preferrable to "\beta_1". It seems very straightforward to understand that "Lane 1" in these tables is referring the coefficient estimates for Lane 1.

Minor comments:
1) To the best of my knowledge, the PLOS One Guidelines for authors require that the references in the text are reported by numbers, and footnotes are not permitted. Please, correct.

Response: thank you for pointing this out, I have made these changes.
