# Supplementary material for: Are there lane advantages in track and field?
Source: PLoS One. 2022 Aug 3;17(8):e0271670. doi: 10.1371/journal.pone.0271670 (PMC9348673; doi:10.1371/journal.pone.0271670)
Supplement: Attachment — Submitted filename: PLOS ONE Response to reviewer comments RR.pdf [file pone.0271670.s005.pdf]

Response to Reviewers:

Reviewer #1

Thank you for your positive assessment of my original manuscript and helpful comments. I hope my responses and changes to the manuscript were satisfactory.

Reviewer #3

Thank you for your time and effort in reviewing my manuscript along with your helpful suggestions. Below I've responded to each of your comments and detailed the changes made to the paper in bold below each.

The paper answers an interesting and "fanciful" question about an alleged benefit in racing in the middle lanes. The paper is well motivated and the results are likely to have a great echo in non-academic fields. The econometric analysis is correctly conducted, and the results obtained seem sound. Notwithstanding, there are some points that deserve to be refined. In particular:
1) notation of eq (1) and (2). As far as I've understood, the dummies L_k represent the Lane k for runner i in heat j and their value is supposed to change according to the runner and to the heat. Therefore, they should also report the subscript ij and the summation is up to k-1 given the collinearity problem.
Response: Thank you for pointing this out, I have made these suggested changes. I modified the summation up to n-1, as the notation should be distinct from the index variable k.

2) When the pulled sample is used a gender dummy should be included

Response: Thanks for this suggestion. A gender dummy wasn't included originally because Personal and Seasonal Bests should pick up a lot of the gender differences. But there certainly could be added information controlled for with a gender dummy, so I have included it in the pooled regressions. The general conclusions from the regressions don't change. If anything, the dummy tightens up some of the standard errors in the pooled regressions, and some of the results are slightly stronger. Again, thanks for this suggestion.

3) Quoting from page 7 "only lane 1 has a statistically significant relationship with SB, which, again, is likely an effect of the low number of observations". It is difficult to understand this claim, it seems the other way round. When the number of observations increases, the standard error (SE) decreases, and consequently the t-stat increases as t=beta/SE(beta), therefore when the number of observations is low one over accepts the null of non significant statistical effects, i.e. weak power. I think the claim should be revised and another piece of explanation for that significance should be put forth.

Response: Thanks for these comments. The idea is that with low N, it's more likely to have Type-I error (erroneously rejecting the null of no effect). I have added a note about this and a reference to Leppink et al. 2016. The pooled results for the randomization checks have changed to some degree with the gender dummy, so lane 1 is no longer significant. In the 100m, only lane 4 is significant, however, with multiple lanes/hypothesis tests, a more appropriate test is to examine if the lane assignments are jointly significant. As such, to strengthen these randomization checks, I have also added F-tests to examine the joint significance of the lanes, and by and large they fail significance at 5% level. In only the women's 100m, F-tests are statistically significant, but they fail 5% significance when the data is pooled with the men's results. In the 200, 400 and 800m all the F-test results are highly

insignificant. These results suggest that, collectively, the lane assignments are unrelated to the prior performance of runners, i.e. randomly assigned.

4) Power problems. I wonder whether there are other tests that can be implemented to analyse the issue whether the results are driven by low power or whether they can be read as pure lack of statistical significance. I am not an expert of this specific filed, but one idea could be to adapt the tests proposed by Cattaneo Titiunik and Vazquez-Bera (2019) to the case under scrutiny.

Response: Thanks for this suggestion. I looked at the suggested paper, and it's tailored to regression discontinuity research designs, which, unfortunately, are a different identification strategy than I take. I'm not an expert with power calculations, but I did some more research and it seems like reporting MDEs, which I do in the paper, is a common approach to discussing ex-post statistical power (e.g. Mckenzie and Ozier 2019). I have added more discussion and a citation motivating the use of MDEs.

Minor issues
The references must be reported according to the common practice followed in the literature. In the current version, no contribution reports the year.

Response: Reporting the years is also the practice I'm familiar and comfortable with, however the PLOS ONE guidelines state: "References are listed at the end of the manuscript and numbered in the order that they appear in the text. In the text, cite the reference number in square brackets (e.g., "We used the techniques developed by our colleagues [19] to analyze the data")."

References
Cattaneo M-D., Titiunik R., and Vazquez-Bera G., 2019. Power calculations for regression-discontinuity designs. The Stata Journal, 19(1): 210-245.

References:

Leppink, Jimmie, Kal Winston, and Patricia O'Sullivan. "Statistical significance does not imply a real effect." ***Perspectives on medical education*** **5.2 (2016): 122-124.**

McKenzie, David, and Owen Ozier. "Why ex-post power using estimated effect sizes is bad, but an ex-post MDE is not." ***World Bank Development Impact Blog*** **(2019).**

Reviewer #2:
I appreciate your time and effort in reviewing my manuscript. Please see my responses to your comments below.

As already stated in my previous report, the topic of the paper is undoubtedly interesting and appealing. However, I still remain with the concerns included in my previous report, mainly due to the fact that almost all major comments/suggestions I made to improve the paper are not taken into account. The statistical methodology applied in the paper is still not clearly described in a general and rigorous way; the statistical models are not reported accurately, for instance formulas (1) and (2). From some author's clarifications, I was able to understand that the author deals with a causal inference framework, but its general and clear description is missing, as well as the relevant literature in this field. Following, the details of the review is reported into major comments.
Major comments:

1. The general description of the statistical methodology applied in the paper is still completely missing. The point is that this cause a misunderstanding of the applied statistical methodology, and it does not allow to appropriately evaluate the validity of the results reported in the paper. From what I was able to understand from the author's corrections made in the manuscript, and some responses, the author deals with a causal inference framework, rather than with classical linear regression model. Regarding on the field one works, in my opinion, the causal inference framework the author deals with, is anything but "unnecessarily/obvious to discuss the theory/assumptions underlying the regression analysis." I wish also to point out that it is not just a regression analysis, but a causal inference framework which is something different from classical linear regression modelling. I still suggest to the author to carefully describe it in details in a separate section, by reporting in a rigorous way the main theory (formulas, assumptions, and also the most relevant literature in the causal inference framework which is completely missing). Regarding the statistical models in formula (1) and (2), they are still reported inaccurately: 〖Time〗_(i,j) to indicate the response variable? Statistical models should be reported accurately in general, for instance using y_ij for the response variable. What about the subscripts: i=1,…?,j=1,…? Also, some statistical terminology: for instance, along the paper just using "specification" along the paper to refer to a statistical model?

Response: Thanks for these comments. I had a hard time interpreting what is being requested here as there are no specific references to relevant literature. The identification strategy is random assignment to treatment. In this case, understanding causal effects amounts to reported average treatment effects. While I implement a regression-based approach, all the regressions are doing is computing the difference in average times by lane number. One could just compute raw means and do this, but the regression framework is convenient because it allows me to control for other correlates, which helps improve precision. In an attempt to respond to the request for more discussion of causal inference, I have added some discussion about what the random assignment buys you (i.e. the equivalence of characteristics of the treatment groups). Thank you for the suggestion regarding subscripts. Following your suggestion and one from the other reviewer I have made some modifications to the notation. I have also changed the language to "regression specification" to avoid any confusion.

2. Again, it is well-known that we cannot speak about a causal effect when considering the "classical" linear regression model. Correlation does not imply causality! Using OLS regression to estimate average treatment effects with random assignment is a causal inference framework, not a "classical" linear regression modelling. This is also why I kindly invite the author to describe briefly describe in a rigorous way the applied statistical methodology, in order to make the paper clear for potential readers.

Response: Thanks for these comments. However, I respectfully disagree. The word "classical" simply refers to the case when the assumptions of OLS are met. There is nothing inherently wrong with using regression for causal inference *if* you are confident that assignment to treatment is random. This is the whole point of the paper-- to leverage the random assignment to lanes to estimate a causal effect. In other words, OLS is simply being used as a statistical method to test a null hypothesis of differences in the data that are generated from random variation. For more discussion on using regression to estimate causal treatment effects with random assignment see Ch. 9 of Gelman and Hill (2006). I have added some discussion about random assignment and causal inference to help clarify the identification strategy.

3. Being revealed that the author use a causal inference framework, and not just a classical linear regression modelling, I have a question. More precisely, the random assignment assumption is

crucial for the validity of the results. The author check such assumption through the statistical model in formula (2): do you think that this is enough to confirm it, and why? What about existing approaches in the literature to deal with this issue; for instance, just to mention one (i.e., not limited to), the propensity score approach? Furthermore, in Section 3.1, in the sentence "…only lane 1 has a statistically significant relationship with SB, which, again, is likely an effect of the low number of observations." Why it should be due to "an effect of the low number of observations"?

Response: Thanks for these comments. I'm sympathetic to the concern about random assignment to treatment, this is an important assumption. In terms of why we should believe it: as stated in the paper, it is in the competition rulebook of the IAAF. It is, of course, possible that they don't follow the rules, so the randomization checks were done as an attempt to confirm adherence to this rule, and they are generally supportive. To conduct them, I look at a runners Season's Best listed in the startlist for each heat. This is the only observable information on the runners I have available (besides gender, but I also group gender separately). Propensity score matching is useful when you are exploring differences across a number of characteristics. But, unfortunately, I only have one characteristic. For the question at hand though -- how race times vary by lane -- SB is arguably the most relevant characteristic as it proxies very well for a runner's ability. To strengthen the randomization check results I have also added F-tests to examine the joint significance of the lanes. In terms of the low number of observations, the concern is that with smaller sample sizes it's more likely to have Type-1 error (incorrectly rejecting the null of no effect). I have added a note about this and a reference to Leppink et al. 2016.

4. Regarding the results for the "pooled" data: why a covariate for gender is not included in the statistical models? How results change if you include also "gender" as a covariate in an appropriate way?
Response: Thanks for this comment, I have added gender as a control in the pooled regressions.

5. Again, in my opinion, suitable models diagnostics should be performed in order to appropriately evaluate the estimated statistical models. They are not just limited to evaluate the "functional form". Just a clarification: the homoscedasticity assumption relates to the error component, and not to the "main variables of interest (lane effects)".
Response: Thanks for these comments. As noted in the results tables, the standard errors reported are all heteroscedasticity-consistent (i.e. robust standard errors).

6. Again, Tables no.1-no.8: in all the tables, the estimated coefficients are reported incorrectly as Lane 1, Lane 3, Wind, etc. For example, the author should to write $\hat{\beta}_1$ rather than Lane 1, $\hat{\beta}_3$ rather than Lane 3, $\hat{\alpha}_1$ rather than Wind, and so on. It could seem very straightforward to understand, but the problem is that this is not correct, and it's an error.

Response: Thanks for these comments. I'm sympathetic to your point, yet the norm is to label the coefficients estimates with the names of independent variables, and not, e.g., \beta_1. Indeed, this is how statistical software (e.g. R, Stata, etc.) reports regression results. I think it's important to follow the norm, so I have left the labelling as is. This practice also avoids the need for readers to keep referring back and forth between to the regression specification and the results tables to understand what each \beta represents.

References:

Gelman, Andrew, and Jennifer Hill. *Data analysis using regression and multilevel/hierarchical models*. Cambridge university press, 2006.
